# Lymphocyte Doubling Time As A Key Prognostic Factor To Predict Time To First Treatment In Early-Stage Chronic Lymphocytic Leukemia

**DOI:** 10.3389/fonc.2021.684621

**Published:** 2021-08-02

**Authors:** Fortunato Morabito, Giovanni Tripepi, Riccardo Moia, Anna Grazia Recchia, Paola Boggione, Francesca Romana Mauro, Sabrina Bossio, Graziella D’Arrigo, Enrica Antonia Martino, Ernesto Vigna, Francesca Storino, Gilberto Fronza, Francesco Di Raimondo, Davide Rossi, Adalgisa Condoluci, Monica Colombo, Franco Fais, Sonia Fabris, Robin Foa, Giovanna Cutrona, Massimo Gentile, Emili Montserrat, Gianluca Gaidano, Manlio Ferrarini, Antonino Neri

**Affiliations:** ^1^Department of Onco-Hematology Azienda Ospedaliera (AO) Cosenza, Biotechnology Research Unit, Cosenza, Italy; ^2^Department of Hematology and Bone Marrow Transplant Unit, Augusta Victoria Hospital, Jerusalem, Israel; ^3^Centro Nazionale Ricerca Istituto di Fisiologia Clinica (CNR-IFC), Research Unit of Reggio Calabria, Reggio Calabria, Italy; ^4^Division of Hematology, Department of Translational Medicine, University of Eastern Piedmont, Novara, Italy; ^5^Hematology, Department of Translational and Precision Medicine, ‘Sapienza’ University, Rome, Italy; ^6^Department of Onco-Hematology AO Cosenza, Hematology Unit AO of Cosenza, Cosenza, Italy; ^7^Mutagenesis and Cancer Prevention Unit, IRCCS Ospedale Policlinico San Martino, Genoa, Italy; ^8^Division of Hematology, Policlinico, Department of Surgery and Medical Specialties, University of Catania, Catania, Italy; ^9^Hematology, Oncology Institute of Southern Switzerland, Bellinzona, Switzerland; ^10^Molecular Pathology Unit, IRCCS Ospedale Policlinico San Martino, Genova, Italy; ^11^Department of Experimental Medicine, University of Genoa, Genoa, Italy; ^12^Hematology Unit, Fondazione IRCCS Ca’ Granda, Ospedale Maggiore Policlinico, Milan, Italy; ^13^Department of Hematology, Hospital Clinic, Institut d’Investigacions Biomèdiques August Pi i Sunyer (IDIBAPS), Barcelona, Spain; ^14^Department of Oncology and Hemato-Oncology, University of Milan, Milan, Italy

**Keywords:** CLL, prognosis, lymphocyte doubling time, TTFT, early stage

## Abstract

The prognostic role of lymphocyte doubling time (LDT) in chronic lymphocytic leukemia (CLL) was recognized more than three decades ago when the neoplastic clone’s biology was almost unknown. LDT was defined as the time needed for the peripheral blood lymphocyte count to double the of the initial observed value. Herein, the LDT prognostic value for time to first treatment (TTFT) was explored in our prospective O-CLL cohort and validated in in two additional CLL cohorts. Specifically, newly diagnosed Binet stage A CLL patients from 40 Italian Institutions, representative of the whole country, were prospectively enrolled into the O-CLL1-GISL protocol (clinicaltrial.gov identifier: NCT00917540). Two independent cohorts of newly diagnosed CLL patients recruited respectively at the Division of Hematology in Novara, Italy, and at the Hospital Clinic in Barcelona, Spain, were utilized as validation cohorts. In the training cohort, TTFT of patients with LDT >12 months was significantly longer related to those with a shorter LDT. At Cox multivariate regression model, LDT ≤ 12 months maintained a significant independent relationship with shorter TTFT along with *IGHV* unmutated (*IGHV*unmut) status, 11q and 17p deletions, elevated β2M, Rai stage I-II, and *NOTCH1* mutations. Based on these statistics, two regression models were constructed including the same prognostic factors with or without the LDT. The model with the LTD provided a significantly better data fitting (χ^2^ = 8.25, P=0.0041). The risk prediction developed including LDT had better prognostic accuracy than those without LDT. Moreover, the Harrell’C index for the scores including LDT were higher than those without LDT, although the accepted 0.70 threshold exceeded in both cases. These findings were also confirmed when the same analysis was carried out according to TTFT’s explained variation. When data were further analyzed based on the combination between LDT and *IGHV* mutational status in the training and validation cohorts, *IGHV*unmut and LDT>12months group showed a predominant prognostic role over *IGHV*mut LTD ≤ 12 months (P=0.006) in the O-CLL validation cohort. However, this predominance was of borden-line significance (P=0.06) in the Barcelona group, while the significant prognostic impact was definitely lost in the Novara group. Overall, in this study, we demonstrated that LDT could be re-utilized together with the more sophisticated prognostic factors to manage the follow-up plans for Binet stage A CLL patients.

## Introduction

The heterogeneous course and outcome of chronic lymphocytic leukemia (CLL) are associated with clinical and laboratory parameters as well as the molecular and cytogenetic complexity of the leukemic clone can contribute to setting a prognosis (1–31). In the past, attention was centered primarily on the predictors of general outcome, intended as overall survival. In contrast, more recently, the focus also has included the definition of time to first treatment (TTFT). According to the current guidelines (32, 33), CLL is treated at progression. Many patients and clinicians often see the start of therapy as a partition between a healthy and a disease condition. Besides, an accurate prediction of the TTFT permits setting the appropriate follow-up strategy. Several methodologies have been proposed to predict the patient’s course and outcome, as well as the TTFT. Although the availability of a battery of cellular/molecular markers has opened the way to an always more refined prognostic stratification of patients, the current practice’s reality indicates difficulties in carrying out several sophisticated features, often confined to a research setting (3). Thus, simplifications introducing inexpensive tests suitable for the clinical setting would be more than welcome, and such prognostic indexes would have a high likelihood of broad applicability.

The breakthrough of novel biologic variables has led to several prognostic indexes to weigh TTFT in early-stage (Binet A) CLL patients (1, 2, 34–52). Indeed, over the past few years, there has been a great effort to use novel molecular markers in prognostic modeling. Yet, questions about their usefulness to improve clinical prediction have been recently debated (53). On the other hand, as the concern for novel molecular markers detected by cutting-edge technologies has soared, performance measures for statistically quantifying their prognostic added value have risen accordingly (52).

The German study group (53) has recently established the independent prognostic value of lymphocyte doubling time (LDT) in Binet stage A patients after more than 50 years following the recognition of a correlation between the lymphocyte proliferation pattern and clinical outcome in CLL (54). LDT, defined as the period needed for the peripheral blood lymphocyte count to reach a double value of that corresponding to the initial observation, is a simple parameter that is useful in arriving at an accurate prognosis in CLL. Whereas a high LDT (greater than 12 months) identifies a population with an excellent prognosis, a low LDT (less than or equal to 12 months) LDT predicts rapid disease progression in patients in the early clinical stages (53, 55, 56). The raising question is whether the re-introduction of LDT among the last generation prognostic factors could help determine the follow-up strategies for an early-stage patient, possibly improving or maintaining the prediction power of more hi-tech markers such as the *IGHV* gene status (51, 57).

Herein, we investigated the LDT predictive value for TTFT in our prospective O-CLL cohort. The results of LDT prognostication power in the O-CLL training cohort were validated in two additional CLL cohorts.

## Materials and Methods

### Lymphocyte Doubling Time

LDT was evaluated at diagnosis, also utilizing lymphocyte counts antecedent the enrollment, if available, and defined as the time needed for the peripheral blood lymphocyte count to reach a value double that of the initial observation (52, 55, 56). LDT was calculated as reported by Hoechstetter et al. (52) through a linear regression based on four blood lymphocyte measurements, each at an interval of a minimum of four weeks from the precedent one in no more than six months before enrollment.

### O-CLL Training Cohort

Newly diagnosed CLL patients from 40 Italian Institutions were prospectively enrolled within 12 months of diagnosis into the O-CLL1-GISL protocol (clinicaltrial.gov identifier: NCT00917540). The ethics committees from each participating center approved this study. Informed consent was obtained from all subjects. Recruitment began in January 2007 and terminated in January 2012. According to the guidelines (32, 33), treatment was decided uniformly for all participating centers based on documented progressive and symptomatic disease. The present analysis was carried out in 498 out of 523 accrued cases where LDT was available.

All patients from the O-CLL cohort were studied for CD38, and ZAP-70 expression, *IGHV* mutational status, FISH assays, and *NOTCH1* and *SF3B1* gene mutations as previously described (18, 28, 29, 58, 59).

The contribution of the single institutions of the training cohort is shown in **Supplementary Table 1**.

### Validation Cohorts

An independent cohort of newly diagnosed and prospectively followed CLL patients recruited since 2001 at the Division of Hematology, Department of Translational Medicine, UPO, Novara, Italy, was utilized as a first validation cohort. The present analysis was restricted to 276, with LDT available, out of 283 cases included in a previous paper (5). All the prognostic factors required in this study (*IGHV* mutational status, Rai stage, β2M, 17(p) and 11(q) deletions, *NOTCH1* coding gene mutation, and LDT were available for 257 cases.

A further independent cohort of newly diagnosed and prospectively followed CLL patients recruited since 2001 at Hospital Clinic, Institute of Hematology and Oncology, University of Barcelona, Spain, was utilized as an additional validation cohort. The present analysis was performed in 414 cases; 355 were included in a recent paper (51). All the prognostic factors required in this study (*IGHV* mutational status, RAI stage, β2M, 17(p) and 11(q) deletions, *NOTCH1* coding gene mutation, and LDT) were available for 247 cases.

### Statistical Analysis

TTFT analyses (including the identification of risk factors for this endpoint) were performed using the Kaplan-Meier method followed by log-rank test. The prognostic impact of specific risk factors on the outcome variable was investigated by univariate and multiple Cox regression analysis. Results are expressed as hazard ratios (HR) and 95% confidence intervals (CI). The main prognostic factor in our study was the _b_Score. It was calculated by deriving a weight corresponding to the regression coefficients of each prognostic factor (b) (60). The regression coefficients of the independent prognostic factors were preliminarily summed up. Then, they were divided by this sum and multiplied by 100, thus deriving a weight ranging from 0 to a given percentage, These weights were summed up on an individual basis, thus deriving a score interpretable in a prognostic scale ranging from 0 to 100% (for patients exposed to all risk factors) (60). The predictive accuracy of the prognostic models was quantified by calculating the Harrell C-index (HC-index), ranging from 0.5 to 1.0, the explained variation on the outcome (i.e., an index combining calibration and discrimination) (61), and the Akaike weights (AIC) (62). The integrated discrimination improvement (IDI) (53) was also calculated to assess the gain in prognostic accuracy provided by LDT. IDI is an index of risk re-classification that quantifies whether a new variable offers a clinically relevant improvement in prediction beyond and above provided by a model based on a previous risk prediction rule and not including the same variable.

The fittings between two nested prognostic models (including and not including LDT) were compared by the -2 log likelihood statistics (63). Data analysis was performed by STATA for Windows v.9 and SPSS Statistics v.21.

## Results

### O-CLL Training Cohort

**Figure 1:**
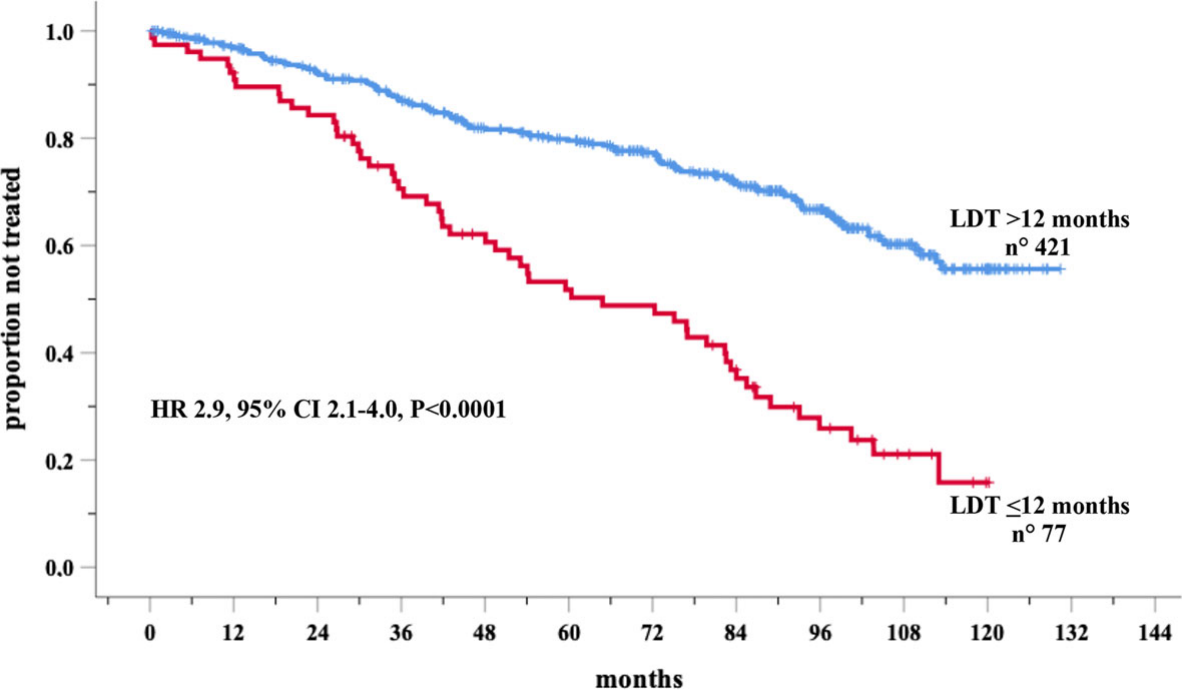
Kaplan-Meir curves of time to first treatment (TTFT) of patients stratified by lymphocyte doubling time (LDT) in the O-CLL training cohort.

**Figure 2:**
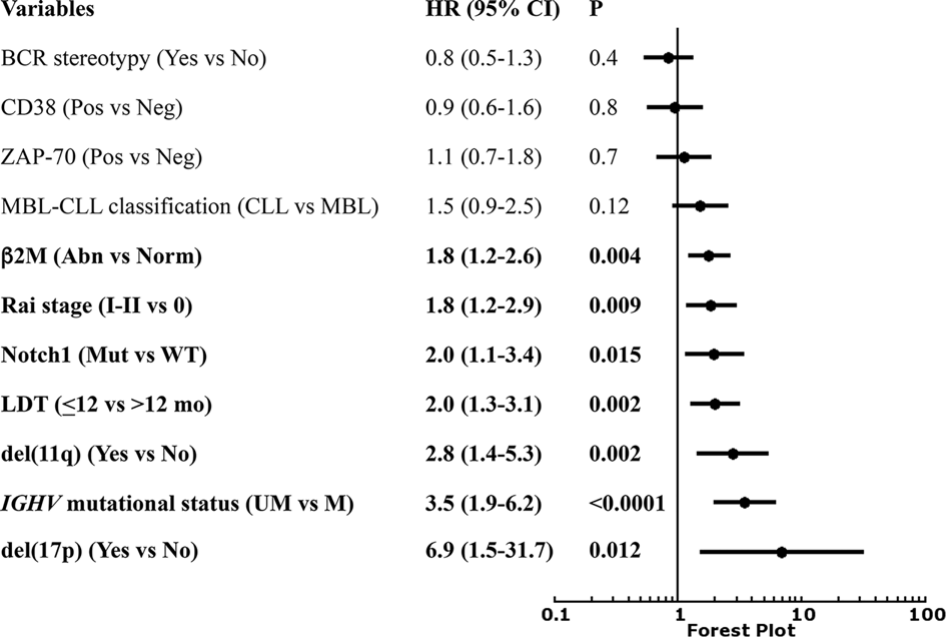
Forest plot of Cox multivariate analysis for time to first treatment (TTFT) according to several variables.

In the prospective O-CLL cohort, LDT was available in 498 Binet stage A CLL cases (median 72.4 months). Seventy-seven cases (15.5%) presented a LDT ≤ 12 months. Out of the 498 patients, 177 needed treatment, with a significantly higher percentage (P<0.0001) of cases requiring therapy detected among the group with LDT ≤ 12 months (54/77, 70.1%) as compared with that with LDT>12 months (123/431, 29.2%). TTFT of patients with LDT ≤12 months was significantly shorter (HR=2.9, 95% CI 2.1–4.0, *P*<0.0001) compared to those with a longer LDT (**Figure 1**). In the same cohort, when all the correlates of outcome determined by univariate analysis (**Table 1**) were introduced into the same multiple Cox regression model, only *IGHV* unmutated (*IGHV*umut) genes, 11q and 17p deletions, elevated β2M, Rai stage I-II, *NOTCH1* mutations and LDT ≤ 12 months maintained a significant independent relationship with shorter TTFT (**Figure 2**).

**Table 1:** Cox univariate analyses of several variables that significantly predict TTFT in the training O-CLL cohort.

Variables	HR (95% CI)	P
Age, >65 years	0.9 (0.6-1.3)	0.5
Rai, I-II stage	1.9 (1.4-2.6)	<0.0001
MBL-CLL classification, CLL	2.3 (1.8-3.4)	<0.0001
SFB1, mutated	2.4 (1.2-4.8)	0.01
Notch1, mutated	2.4 (1.7-3.5)	<0.0001
β2-M, abnormal	2.2 (1.5-3.1)	<0.0001
ZAP-70, positive	2.8 (2.1-3.8)	<0.0001
CD38, positive	3.2 (2.3-4.3)	<0.0001
*IGHV* status, unmutated	5.4 (3.9-7.3)	<0.0001
BCR stereotypy, yes	1.7 (1.1-2.4)	0.002
Fish analysis, 11q deletion	5.3 (3.5-8.1)	<0.0001
Fish analysis, 17p deletion	5.2 (2.4-11.2)	<0.0001

TTFT, time to first treatment; HR, hazards ratio; 95% CI, 95% confidence interval; β2M, β2-microglobulin; ULN, upper limit of normal.

Starting from this analysis, two regression models were constructed, i.e., a Cox model including all significant and independent correlates of TTFT except LDT (Model 1, **Table 2**) and a Cox model including the same set of prognostic factors and LDT (Model 2, **Table 2**). Of note, Model 2 (including LDT) provided a significantly better data fitting (χ^2 =^ 8.25, P=0.0041) than Model 1 (not including LDT). Moreover, by IDI calculations, we demonstrated that LDT increased the estimated risk of +3.8%, a result of high statistical significance (P<0.001). These analyses were carried out in 334 cases in which all the variables were available.

**Table 2:** Regression coefficients (b) derived from two multivariate models where lymphocyte doubling time (LDT) where excluded (_NO_LDT MODEL) or included (LDT MODEL).

Variables	_NO_LDT MODEL (Model 1)			LDT MODEL (Model 2)		
	b	HR (95% CI)	P	b	HR (95% CI)	P
del(17p) (Yes *vs* No)	2.225	9.3 (2.1-40.1)	0.003	1.921	6.8 (1.6-29.8)	0.011
*IGHV* mutational status (UM *vs* M)	1.279	3.6 (2.4-5.4)	<0.0001	1.271	3.6 (2.5-5.4)	<0.0001
del(11q) (Yes *vs* No)	1.025	2.8 (1.5-5.1)	0.001	0.988	2.7 (1.5-4.9)	0.002
β2M (Abn *vs* Norm)	0.662	1.9 (1.3-2.8)	<0.0001	0.668	1.9 (1.4-2.8)	<0.0001
Rai stage (I-II *vs* 0)	0.734	2.1 (1.4-3.1)	<0.0001	0.628	1.8 (1.3-2.8)	0.002
*NOTCH1* gene (Mut vs WT)	0.576	1.8 (1.1-2.9)	0.023	0.551	1.7 (1.1-2.8)	0.03
LDT (≤12 *vs* >12 mo)	…	…	…	0.604	1.8 (1.4-2.8)	0.003

These analyses were carried out in 334 cases of the O-CLL training cohort in which all the variables were available.

### Prediction Risk Scores in Training O-CLL Cohort

**Figure 3:**
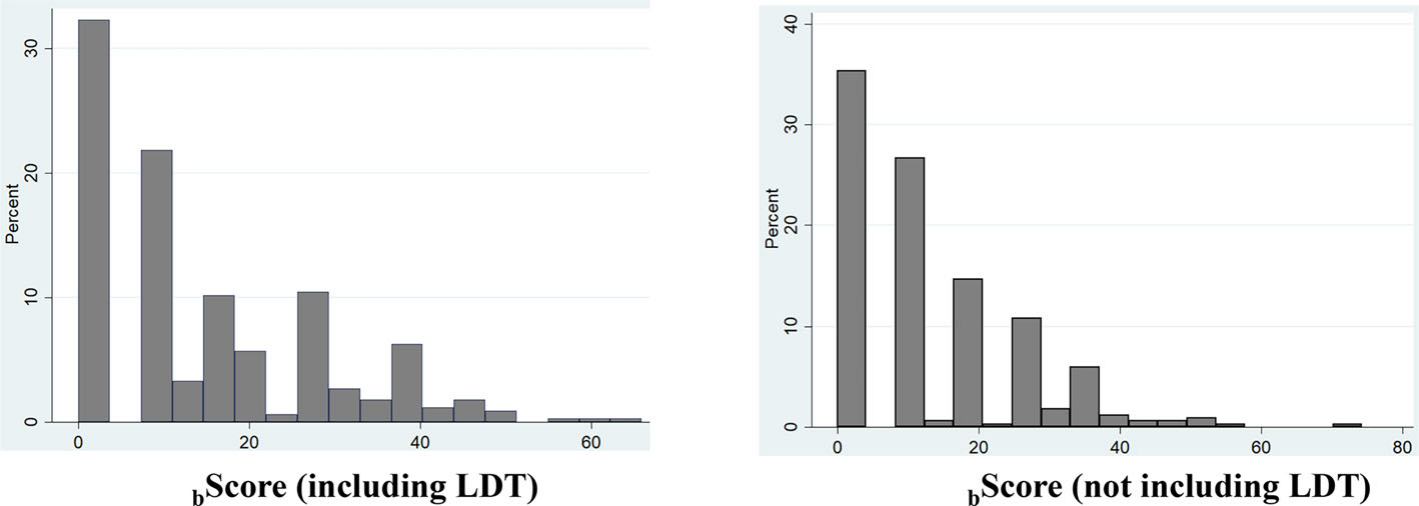
Distribution of bScore with or without Lymphocyte Doubling Time (LDT).

Based on Model 1 and Model 2, two risk prediction rules were developed, i.e. _b_Score LDT and _b_Score no LDT (**Figure 3**). _b_Score which included LDT, had better prognostic accuracy than that without LDT (**Table 1**, training set). Moreover, the HC-index including LDT, was higher than those without LDT (75.4 *versus* 74.7), although the accepted 0.70 threshold (25) exceeded in both cases (**Table 3**). This was also true when the same analysis was carried out according to the explained variation in TTFT, which combines the discrimination and the calibration abilities of a risk prediction rule (_b_Score LDT = 47.6% *versus*_b_Score no LDT = 45.0%; **Table 3**).

**Table 3:** Comparison between the _NO_LDT score and the LDT score for the prediction of TTFT in the O-CLL, in the O-CLL, Novara, and Barcelona cohorts by regression coefficients (_B_Score).

*O-CLL cohort (training set)*	_b_SCORE	
	LDT	_NO_LDT
**HC index (%)**	75.4	74.7
**Explained variation (%)**	47.6	45.0
**Akaike weights (%)**	98.1	1.9
***Novara cohort (validation set)***		
**HC index (%)**	80.4	75.2
**Explained variation (%)**	49.6	33.3
**Akaike weights (%)**	100%	0%
***Barcelona cohort (validation set)***		
**HC index (%)**	70.0	68.1
**Explained variation (%)**	30.5	25.8
**Akaike weights (%)**	98.2	0.8

### Prediction Risk Scores in Validation Novara and Barcelona Cohorts

**Figure 4:**
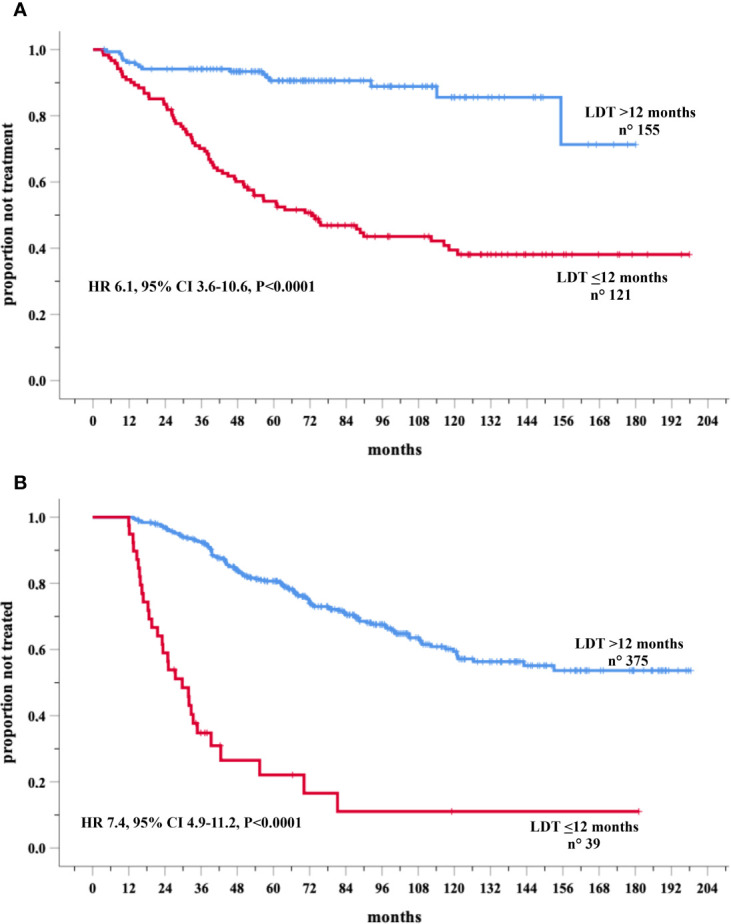
Kaplan-Meir curves of time to first treatment (TTFT) of patients stratified by lymphocyte doubling time (LDT) in the Novara **(A)** and Barcelona **(B)** validation cohorts.

These analyses were carried out in two validation cohorts. LDT was available in 276 and in 414 Binet stage A cases respectively in Novara and Barcelona cohorts. Eighty-six and 148 patients required therapy in the Novara and Barcelona cohorts, respectively. A significantly higher percentage of treated cases were recorded among the group with LDT ≤ 12 months in both the Novara (LDT ≤ 12 70/121, 57.9% *versus* LDT>12 months (16/155, 10.3%) and the Barcelona (LDT ≤ 12 months 30/39, 76.9% *versus* LDT>12 months cohort 119/375, 31.7%). In the Novara cohort, Cox univariate analysis showed a significantly increased risk of treatment for patients with LDT ≤12 months (155 cases) (HR=6.1, 95% CI 3.6–10.6, *P*<0.0001) compared to those with a longer LDT (121 patients) (**Figure 4A**). Similar results were detected in the Barcelona validation set, in which the 39 cases with LDT ≤12 months showed a risk of being treated 7.4 times higher (95% CI 4.9–11.2, *P*<0.0001) than the 375 cases with a longer LDT (HR=1) (**Figure 4B**).

**Table 4** reports the clinical and biological variables significantly associated with TTFT in the multivariate models of training as well as both validation cohorts. Remarkably, the higher prognostic value, provided by risk prediction rule including LDT, found in the training cohort was fully confirmed in the two validation cohorts (**Table 3**). The HC-indexes and the explained variations in TTFT were consistently higher for bScore including LDT than for that excluding this variable (**Table 3**) in both Novara cohort (HC-Indexes 80.4 *versus* 75.2; explained variations 49.6 *versus* 33.3) and Barcelona cohort (HC-Indexes 70.0 *versus* 68.1; explained variations 30.5 *versus* 25.8). Finally, the Akaike weights coherently indicated that the risk scores including LDT had a chance to provide the best prognostic estimates ranging from 98.1% to 100% in both the training and in the two validation cohorts (**Table 3**).

**Table 4:** Comparison of the clinical and biological variables of O-CLL (training), Novara and Barcelona (validation) cohorts resulted significantly associated to time to first treatment in the multivariate models.

Variables	O-CLL cohort	Novara cohort	Barcelona cohort	Total (%)
	Number of cases (%)	Number of cases (%)	Number of cases (%)	Number of cases (%)
**LDT**	**498 (41.9)**	**276 (23.2)**	**414 (34.8)**	**1188 (100)**
≤12months	77 (15.5)	121 (43.8)	39 (9.4)	237 (19.9)
>12 months	421 (84.5)	155 (56.2)	375 (90.6)	951 (80.1)
***IGHV* mutational status**	**482 (45.2)**	**265 (24.8)**	**320 (30)**	**1067 (100)**
UM	150 (31.1)	67 (25.3)	113 (35.3)	330 (30.9)
M	332 (68.9)	198 (74.7)	207 (64.7)	737 (69.1)
**del(11q)**	**477 (42.4)**	**274 (24.4)**	**373 (33.2)**	**1124 (100)**
Yes	30 (6.3)	15 (5.5)	28 (7.5)	73 (6.5)
No	447 (93.7)	259 (94.5)	345 (92.5)	1051 (93.5)
**β2M**	**343 (34.0)**	**268 (26.6)**	**397 (39.4)**	**1008 (100)**
Abnormal	127 (37.0)	126 (47.0)	158 (39.8)	411 (40.8)
Normal	216 (63.0)	142 (53.0)	239 (60.2)	597 (59.2)
**Rai stage**	**493 (41.7)**	**276 (23.3)**	**414 (35)**	**1183 (100)**
I-II	104 (21.1)	64 (23.2)	80 (19.3)	248 (21.0)
0	389 (78.9)	212 (76.8)	334 (80.7)	935 (79.0)
***NOTCH1* gene**	**487 (46.9)**	**276 (26.6)**	**275 (26.5)**	**1038 (100)**
Mut	63 (12.9)	21 (7.6)	28 (10.2)	112 (10.8)
WT	424 (87.1)	255 (92.4)	247 (89.8)	926 (89.2)
**del(17p)**	**477 (42.4)**	**274 (24.4)**	**373 (33.2)**	**1124 (100)**
Yes	10 (2.1)	9 (3.3)	14 (3.8)	33 (2.9)
No	467 (97.9)	265 (96.7)	359 (96.2)	1091 (97.1)

Cases with all available variables have been reported. All variables are in bold.

### Relationship Between LDT and IGHV Mutational Status

**Figure 5:**
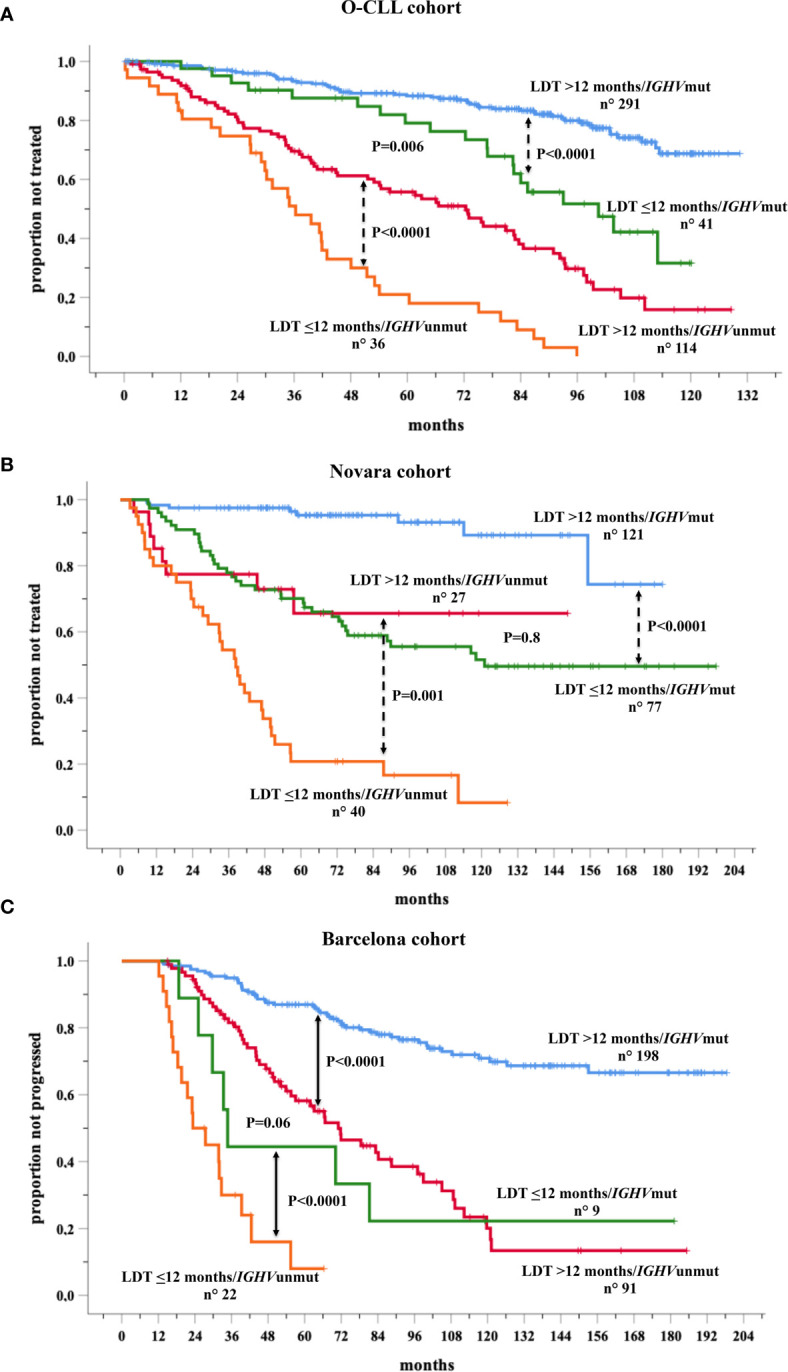
Kaplan-Meir curves of time to first treatment (TTFT) of patients stratified by the combined analysis of lymphocyte doubling time (LDT) and *IGHV* mutational status in the O-CLL training cohort **(A)** and Novara **(B)** and Barcelona **(C)** validation cohorts for whom both variables were available.

Data were further analyzed based on the combination between LDT and *IGHV* mutational status in the training and validation cohorts, in cases with both variables available As expected, LDT ≤12 months significantly maintained its negative prognostic power in both the *IGHV*mut and in the *IGHV*unmut patient groups in O-CLL (training) cohort (**Figure 5A**) as well as in both Novara (**Figure 5B**) and Barcelona (**Figure 5C**) validation cohorts, showing *IGHV*unmt & LDT ≤ 12 months and *IGH*Vmut & LDT>12 months the shortest and the longest TTFT, respectively. Interestingly, the probability of remaining therapy-free at 6 years was 51% in the *IGHV*unmut and LDT>12months group and of 76% in the *IGHV*mut LTD ≤ 12 months, respectively (P=0.006) in the O-CLL validation cohort, confirming the predominant prognostic role of the *IGHV*umut status. However, this predominance was of borden-line significance (P=0.06) in the Barcelona group, while the significant prognostic impact was definitely lost in the Novara group. This discrepancy could be due to a different distribution of the above-mentioned subsets (*IGHV*unmut and LDT>12months and *IGHV*mut LTD ≤ 12 months) among Novara validation cohort (**Supplementary Figure 1**).

## Discussion

The clinical course of early-stage CLL is hugely heterogeneous. While some patients need treatment at the onset of the diagnosis, others remain therapy-free for many years or even do not receive any treatment livelong (1, 2).

Several prognostic algorithms derived from multivariable models, nomograms and score systems have been developed to predict clinical outcomes accurately in early-stage CLL (64). The *IGHV* gene configuration is one of the most important single factors predicting therapy need, and it is recurrently incorporated in all prognostic models (65).

In contrast, the prognostic role of LDT in CLL was acknowledged more than 35 years ago by Montserrat et al. (55) and soon after by Molica et al. (56), when disease biology of the neoplastic cell remained weakly recognized. The raising question is whether the re-introduction of LDT among the last generation prognostic factors could help determine the follow-up strategies for an early-stage patient, possibly improving the prediction power of more sophisticated markers such as *IGHV* gene status. In the prospective O-CLL training cohort, a Cox regression model indicated *IGHV*umut genes, 11q and 17p deletions, elevated β2M, Rai stage I-II, *NOTCH1* mutations and LDT ≤ 12 months as independently associated with shorter TTFT, thus confirmed the prognostic value of LDT in the era of new prognostic indicators. Our results fully agree with those of the German CLL Study Group, which endorses the prognostic benefit of determining LDT in the era of new markers (52). Specifically, the German prognostic model includes genetic features, i.e., 17p and 11q deletions, as well as *IGHV* mutational status. Similarly, the same prognostic indicators were demonstrated by our group to be independently associated with TTFT together with LDT. Unlike CLL1-PM, *NOTCH1* remained significant in our analysis, confirming previous results (3, 5, 18).

Remarkably, the higher prognostic value, provided by risk prediction score including LDT, found in the training cohort was fully confirmed in the two validation cohorts. Specifically, the indicators of performance, such as the HC-indexes and the explained variations, were consistently higher for bScore including LDT than for that excluding this variable in the training and both validation cohorts. Finally, the Akaike weights coherently indicated that the risk scores including LDT had a chance to provide the best prognostic estimates ranging from 98.1% to 100% in both the training and the two validation cohorts. More recently, the prognostic significance of LDT was demonstrated to be independent for TTFT from the CLL-IPI and the Barcelona/Brno prognostic models in a real-life cohort of 848 Binet stage A patients (66).

Thus, the question of whether the re-introduction of LDT among the more sophisticated prognostic factors could help to determine the follow-up strategies for an early-stage patient has an affirmative answer. However, several caveats have to be considered. Although simple, LDT determinations require a precise timing and relative frequent accesses of patients to the clinic, thus preventing the setting of a definite ‘watch and wait’ strategy in concomitance with the work-up at diagnosis. Alternatively, more precise and possibly more rapid methodologies to measure lymphocyte proliferating potential, such as labeling with deuterated water, are too complex to be used routinely (67). Moreover, when data were further analyzed based on the combination between LDT and *IGHV* mutational status in the training and validation cohorts, *IGHV*unmut and LDT>12 months group showed a predominant prognostic role over *IGHV*mut LTD ≤ 12 months (P=0.006) in the O-CLL validation cohort. However, this predominance was of borden-line significance (P=0.06) in the Barcelona group, while the significant prognostic impact was definitely lost in the Novara group, incongruity possibly due to a different distribution of the above-mentioned subsets among Novara validation cohort. Thus, the *IGHV* mutation status could offer some, likely marginal, sensitivity advantage over the LDT determination. Finally, no information on the LDT stability overtime is available. Therefore, LDT values may vary over time, particularly in concomitance with the progression of the CLL towards a more aggressive form. These changes are likely to relate to a minority of cases and may not influence an entire cohort’s data but may have clinical relevance for the individual patients. These variations in time have been reported for cellular markers such as CD38 or ZAP-70 and have caused their subsequent obsolescence.

## Data Availability Statement

The raw data supporting the conclusions of this article will be made available by the authors, without undue reservation.

## Ethics Statement

The studies involving human participants were reviewed and approved by Comitato Etico Regionale Liguria n° OMC07.002. The patients/participants provided their written informed consent to participate in this study.

## Author Contributions

FMo, MG, MF, and AN designed the study. FMo, GT, MG, and GD’A performed statistical analysis. FMo, MG, GT, DR, GG, EMo, MF, GC, MC, FF, FS, SB, SF, and AN analyzed and interpreted data, and wrote the manuscript. All authors contributed to the article and approved the submitted version.

## Funding

Associazione Italiana Ricerca sul Cancro (AIRC) Grant 5 x 1000 n.9980 (to FM, MF, and AN); AIRC and Fondazione CaRiCal co-financed Multi-Unit Regional Grant 2014 n.16695 to FM; AIRC, Special Program Metastases (n. 21198) 5x1000 to RF and GG; RF-2018-12365790, MoH, Rome, Italy (to GG); AIRC IG-5506 to GF, IG-14326 (to MF), IG-15426 (to FF); Compagnia S. Paolo, Turin, Italy, Project 2017.0526 (to GF) and by the Ministry of Health (Project 5x1000, 2015 and 2016 and Current Research 2016 (to GF, GC, and FF); Swiss Cancer League, ID 3746, 4395 4660, and 4705, Bern, Switzerland; European Research Council (ERC) Consolidator Grant CLLCLONE, ID: 772051; Swiss National Science Foundation, ID 320030_169670/1 and 310030_192439, Berne, Switzerland; The Leukemia & Lymphoma Society, Translational Research Program, ID 6594-20, New York; Italian Ministry of Health_Ricerca Corrente 2021 to AN.

## Conflict of Interest

The authors declare that the research was conducted in the absence of any commercial or financial relationships that could be construed as a potential conflict of interest.

The reviewer FA declared a shared affiliation with one of the authors, AN, to the handling editor at time of review.

## Publisher’s Note

All claims expressed in this article are solely those of the authors and do not necessarily represent those of their affiliated organizations, or those of the publisher, the editors and the reviewers. Any product that may be evaluated in this article, or claim that may be made by its manufacturer, is not guaranteed or endorsed by the publisher.
