# Supplementary figures and images for: Lymphocyte Doubling Time As A Key Prognostic Factor To Predict Time To First Treatment In Early-Stage Chronic Lymphocytic Leukemia
Source: Front Oncol. 2021 Aug 2;11:684621. doi: 10.3389/fonc.2021.684621 (PMC8366564; doi:10.3389/fonc.2021.684621)

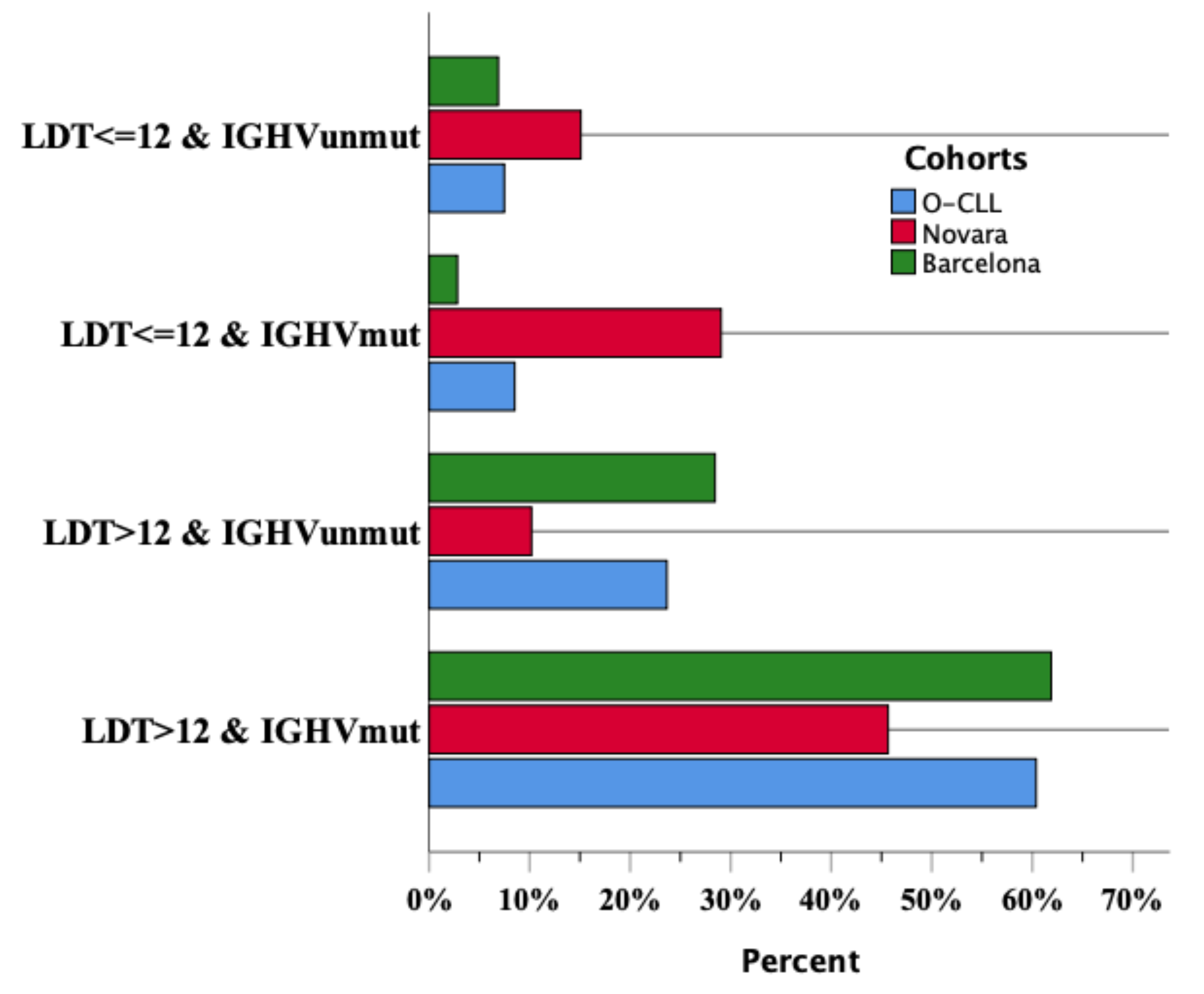

Supplement: Supplementary file 1 [file Image_1.tif]
