# Supplementary material for: Lymphocyte Doubling Time As A Key Prognostic Factor To Predict Time To First Treatment In Early-Stage Chronic Lymphocytic Leukemia
Source: Front Oncol. 2021 Aug 2;11:684621. doi: 10.3389/fonc.2021.684621 (PMC8366564; doi:10.3389/fonc.2021.684621)
Supplement: Supplementary file 2 [file Table_1.docx]

**Supplementary Table 1.** The contribution of the single institutions of the training cohort

| **Partecipating Centers** | **No of enrolled patients** |
| --- | --- |
| Unità di Ematologia e Trapianto di Cellule Staminali, Istituto di Oncologia “Giovanni Paolo II”, Bari | 8 |
| Dipartimento di Ematologia, Ospedale di Venere, Bari | 13 |
| Divisione di Ematologia, Presidio Ospedaliero “A. Perrino”, Brindisi | 9 |
| U.O.C. di Oncoematologia Ospedale “S. Anna e S. Sebastiano”, Caserta | 2 |
| U.O.S. di Emato-Oncologia, Ospedale Garibaldi-Nesima, Catania | 16 |
| Divisione di Ematologia, Università di Catania Ospedale Ferrarotto, Catania | 58 |
| UOC Ematologia, Pugliese-Ciaccio Hospital, Catanzaro | 28 |
| UOC Oncologia, Pugliese-Ciaccio Hospital, Catanzaro | 9 |
| U.O.C di Ematologia, A.O. Cosenza | 28 |
| U.O.C. di Oncologia, Ospedale Giannettasio, Rossano Calabro | 4 |
| Clinica Ematologica, DIMI, Genova | 15 |
| Oncologia medica C IRCCS Ospedale Policlinico San Martino, Genoa, | 3 |
| Ospedale Villa Scassi Sampierdarena, Genova | 3 |
| Ematologia, A.O. San Martino, Genova | 7 |
| Unità di Ematologia, Ospedale Vito Fazzi, Lecce | 24 |
| UOC di Ematologia Ospedale di Matera | 19 |
| Divisione di Ematologia, Ospedale Papardo, Messina | 16 |
| Divisione di Ematologia, Università di Messina | 42 |
| Ematologia and CTMO, Foundation IRCCS Ca’ Granda Ospedale Maggiore Policlinico, Milano | 16 |
| U. O. C. Oncologia ed Ematologia Oncologica, Mirano Venezia | 9 |
| Oncoematologia Policlinico di Modena | 2 |
| Divisione di Ematologia, Ospedale Cardarelli, Napoli | 3 |
| Divisione di Ematologia, Ospedale Policlinico, Palermo | 17 |
| Ematologia, CTMO, Azienda Ospedaliera Universitaria di Parma | 1 |
| Dipartimento di Ematologia, Ospedale Santo Spirito, Pescara | 14 |
| Unità di Ematologia, Dipartimento di Onco-Ematologia, Guglielmo da Saliceto Hospital, Piacenza | 10 |
| Unità di Ematologia, A.O. of Reggio Calabria | 20 |
| Unità Operativa di Ematologia, A.O.S. Maria Nuova, Reggio Emilia | 29 |
| S.C. di Ematologia e Trapianto di Cellule Staminali IRCCS-CROB di Rionero in Vulture, Potenza | 5 |
| Dipartimento di Ematologia, Ospedale Nuovo Regina Margherita, Roma | 3 |
| Ematologia, A.O. Sant’Andrea, Università La Sapienza, Roma | 6 |
| Divisione di Ematologia, Università La Sapienza, Roma | 16 |
| Unità di Ematologia e Trapianto di Cellule Staminali, IRCCS Ospedale Casa Sollievo della Sofferenza, San Giovanni Rotondo | 1 |
| Unità di Ematologia, Ospedale San Vincenzo, Taormina | 9 |
| Unità di Ematologia, Ospedale San Nicola Pellegrino, Trani | 3 |
| Centro di Riferimento Ematologico-Seconda Medicina, Azienda Ospedaliero-Universitaria, Ospedali Riuniti, Trieste | 18 |
| U.O. Oncologia Medica, Ospedale di Circolo Fondazione Macchi, Varese | 3 |
| Unità Operativa di Ematologia, Ospedale dell'Angelo, Venezia-Mestre | 6 |
| Dipartimento di Oncologia, Ospedale Civile, Noale, Venezia | 5 |
